# The role of tumor metabolism in modulating T-Cell activity and in optimizing immunotherapy

**DOI:** 10.3389/fimmu.2023.1172931

**Published:** 2023-04-26

**Authors:** Shonik Ganjoo, Priti Gupta, Halil Ibrahim Corbali, Selene Nanez, Thomas S. Riad, Lisa K. Duong, Hampartsoum B. Barsoumian, Fatemeh Masrorpour, Hong Jiang, James W. Welsh, Maria Angelica Cortez

**Affiliations:** ^1^ Department of Radiation Oncology, The University of Texas MD Anderson Cancer Center, Houston, TX, United States; ^2^ Department of Medical Pharmacology, Cerrahpasa Medical Faculty, Istanbul University-Cerrahpasa, Istanbul, Türkiye

**Keywords:** metabolism, T cell, cancer, immunotherapy, tumor immune microenvironment

## Abstract

Immunotherapy has revolutionized cancer treatment and revitalized efforts to harness the power of the immune system to combat a variety of cancer types more effectively. However, low clinical response rates and differences in outcomes due to variations in the immune landscape among patients with cancer continue to be major limitations to immunotherapy. Recent efforts to improve responses to immunotherapy have focused on targeting cellular metabolism, as the metabolic characteristics of cancer cells can directly influence the activity and metabolism of immune cells, particularly T cells. Although the metabolic pathways of various cancer cells and T cells have been extensively reviewed, the intersections among these pathways, and their potential use as targets for improving responses to immune-checkpoint blockade therapies, are not completely understood. This review focuses on the interplay between tumor metabolites and T-cell dysfunction as well as the relationship between several T-cell metabolic patterns and T-cell activity/function in tumor immunology. Understanding these relationships could offer new avenues for improving responses to immunotherapy on a metabolic basis.

## 1 Introduction

Metabolic reprogramming has historically been recognized as a key hallmark of cancer based on its significance for supporting and maintaining a malignant phenotype (1–3). Because cancer cells must survive in the harsh and often nutrient-depleted conditions of solid tumors, their reprogrammed metabolism and overall metabolic plasticity are necessary to meet the bioenergetic demands needed to sustain growth and proliferation (4, 5). Despite enormous genomic, phenotypic, and molecular heterogeneity across cancer types, altered metabolism continues to be a unifying biological feature of cancer that has consistently been linked with tumor growth (6–8). In the 1920s, Otto Warburg’s observation that tumors could rapidly metabolize glucose and produce lactose, even in the presence of oxygen and functioning mitochondria, marked a seminal discovery in cancer metabolism (9). However, this phenomenon, termed the Warburg effect, has historically been the subject of intense debate. Although several proposals have been raised to explain the Warburg effect, its function remains controversial. Nonetheless, the current understanding of cancer metabolism positions the Warburg effect within a larger set of interrelated processes that provide tumors with a metabolic advantage. These processes include the activation of growth-promoting oncogenes, expression of glucose transporters, and loss of function of tumor suppressors. Cumulatively, these molecular and functional processes accelerate glycolytic flux and production of adenosine triphosphate (ATP), but they also support the biosynthesis of organic molecules, maintain redox homeostasis, and drive immunosuppression (10). Crucially, the evolving understanding of cancer metabolism is also important for highlighting the role that metabolism plays in the broader context of tumor immunology.

The complexity of the tumor immune microenvironment is an especially important obstacle to progress in the field of cancer immunotherapy. Despite revolutionary advancements in immune checkpoint inhibitors (ICIs) to improve antitumor T-cell activity (e.g., anti-PD1 and anti-CTLA4), immunologically “cold” tumors remain challenging to treat. Mounting evidence suggests that altered tumor metabolism has key roles in suppressing T-cell activity and reducing the therapeutic efficacy of ICIs (11–13). Also, the tumor microenvironment (TME) and the broader metabolic demands of tumors can both directly interfere with the normal metabolic profiles of activated T cells, dampening antitumor effector functions (14–16). Several ongoing investigations have targeted specific immunosuppressive metabolites or metabolism-regulating molecules as a step towards generating more potent T-cell effectors as well as lasting memory cells (14). Metabolic interventions can also be beneficial for remodeling the tumor immune microenvironment by exploiting differences in the metabolic demands of T-cell subsets (e.g., regulatory T cells [T_regs_] vs cytotoxic T cells) (17). Hence, understanding the mechanistic intersections of tumor metabolism and T-cell metabolism is especially important for developing more effective immunotherapeutic strategies.

In this Review, we explore the role of metabolism in mediating the dynamic relationship between T cells and tumors. We also discuss the relevance of specific T-cell metabolic pathways for supporting antitumor T-cell function and examine how the TME can fundamentally interfere with these pathways to modulate T-cell activity, including on an epigenetic basis. We further highlight some of the key metabolites present in the TME that promote T-cell dysfunction, which also have potential as therapeutic targets to overcome immunosuppression. Finally, we present some pertinent molecular strategies for targeting metabolism to bolster antitumor activity and consider the clinical significance of adopting strategies that integrate metabolism with immunotherapy to enable long-term antitumor immune activity.

## 2 The dynamic interrelationship between T cells and tumor metabolism

**Figure 1:**
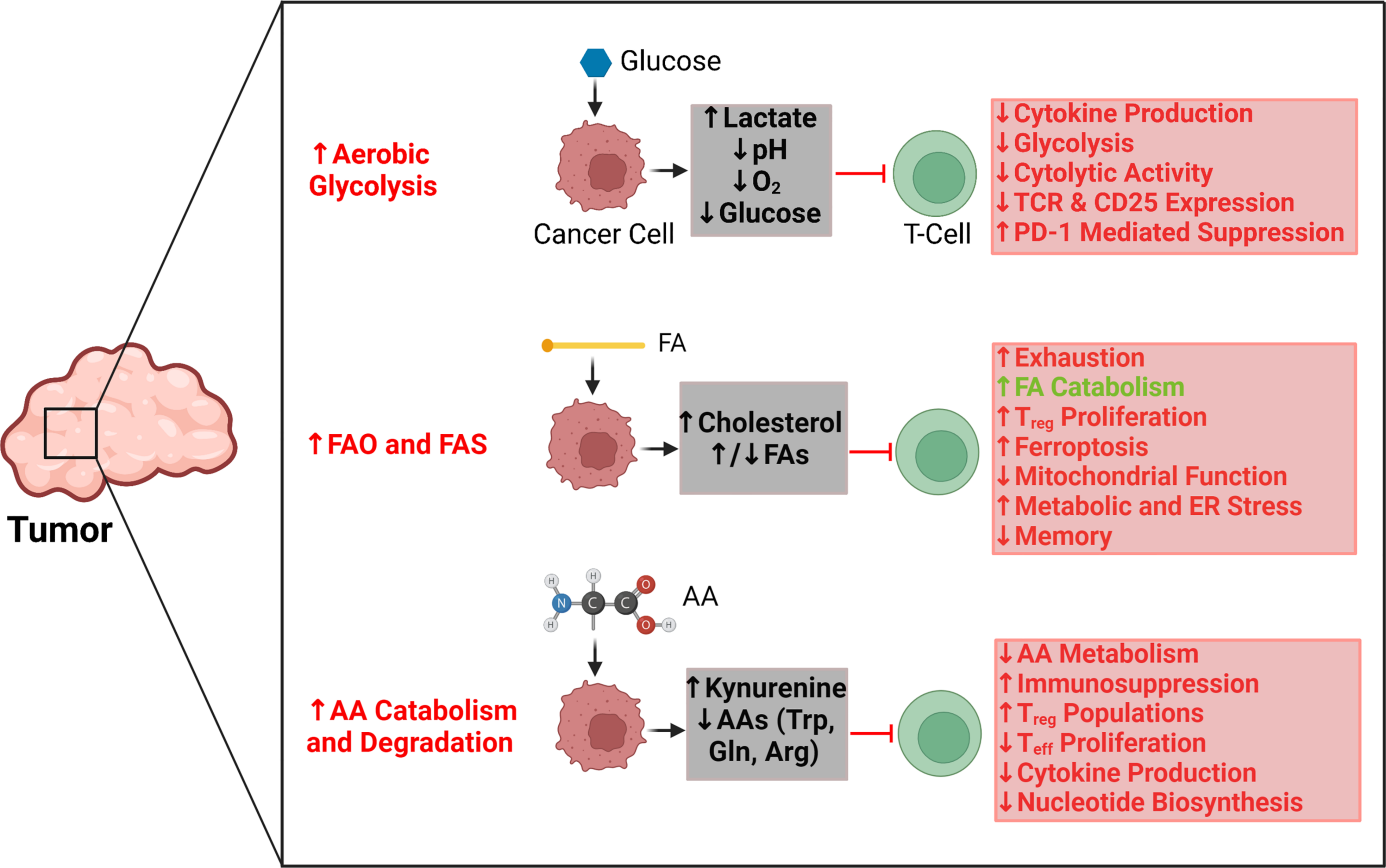
Key regulatory effects and interactions between tumor metabolic pathways and T-cell activity. Aerobic glycolysis in tumors is primarily responsible for creating a hypoxic, acidic, and nutrient-depleted microenvironment that suppresses effector T-cell activity and blunts antitumor immune responses. Similarly, the rapid uptake and catabolism of amino acids by tumors reduces their availability for T cells, which further promotes immunosuppression and supports the metabolic activities of regulatory T cells as opposed to effector T cells. Tumors can upregulate both fatty acid oxidation (FAO) and fatty acid synthesis (FAS), which affect T-cell activity differently. The accumulation of select fatty acids can support FAO in T cells; however, those same fatty acids can also promote exhaustion and inhibit the proliferation of memory T cells. Cholesterol is particularly significant for causing mitochondrial and endoplasmic reticulum (ER) stress in T cells. Red arrows and text indicate tumor metabolism–mediated effects that can suppress antitumor T-cell activity. Green arrows and text indicate tumor metabolism–mediated effects that can support antitumor T-cell activity. AA, amino acid; Arg, arginine; ER, endoplasmic reticulum; FA, fatty acid; FAO, fatty acid oxidation; FAS, fatty acid synthesis; Gln, glutamine; PD1, programmed cell death protein 1; T_reg_, regulatory T cell; T_eff_, effector T cell; TCR, T-cell receptor; Trp, tryptophan.

The independent metabolic profiles of T cells and cancer cells have been extensively characterized (2, 18–20). Indeed, the metabolic patterns of each of these cell types provide important insights into their separate functions and activity. However, the similarities in metabolic demands, pathways, and activities between T cells and cancer cells have drawn significant attention to the dynamic interrelationship between these cells based on metabolism (21). Here, we contextualize this interrelationship by examining the reciprocal interactions between T cells and tumors through three salient metabolic pathways: glycolysis, fatty acid oxidation, and amino acid catabolism (**Figure 1**).

### 2.1 Glycolysis

Glycolysis is a fundamental metabolic pathway used by both T cells and cancer cells as a source of energy to support their various activities (22–24). Notably, T cells exhibit metabolic “switching” depending on their activation status (25). Quiescent T cells primarily rely on oxidative phosphorylation to generate ATP, whereas activated T cells upregulate glycolysis to support proliferation and effector functions (26, 27). Glycolysis can also create byproducts that can be used in other macromolecular biosynthetic pathways, such as glycogenesis or the pentose phosphate pathway (22). Glucose is a critical substrate for promoting the normal activity and functioning of effector T cells, and T-cell cytokine production is fundamentally tied to glycolytic flux as well (27, 28). Tumors show similar metabolic reprogramming, although the shift in tumor metabolism does not require an activating or co-stimulatory signal, as is the case for T cells (29). Tumors uniquely rely on “aerobic glycolysis,” which is the use of glycolysis for energy even under aerobic conditions (30, 31). In addition to upregulating various isoforms of glucose transporters (GLUTs) that facilitate glucose uptake from the environment, cancer cells can also chronically obtain glucose through glycogenolysis and gluconeogenic mechanisms (32–35). Due to the abnormally high glycolytic flux in cancer cells, it is estimated that the glucose concentration is approximately 10-fold lower in the tumor interstitium relative to the plasma (36). Furthermore, the rapid consumption of glucose and its conversion into lactic acid in the TME fulfills the anabolic demands of various cancer cells by quickly generating ATP to support proliferation, regardless of oxygen availability (37, 38). Crucially, the metabolic reprogramming of tumors to rapidly generate ATP from aerobic glycolysis is also directly responsible for creating the acidic, hypoxic, and nutrient-depleted conditions of the TME, which have profound consequences for T-cell activity (39, 40).

#### 2.1.1 Lactic acid accumulation and acidity

One of the most significant consequences of aerobic glycolysis is the acidification of the TME (9, 41). The accumulation of lactic acid and corresponding decrease in pH of the TME has numerous inhibitory effects on the activity and function of tumor-infiltrating lymphocytes (TILs), especially cytotoxic T lymphocytes (CTLs) (15, 40). One study showed that lactic acid inhibited the migratory capacities of CD4+ and CD8+ T cells while also inhibiting the cytolytic activity of CD8+ T cells, which collectively blunted their pro-inflammatory activity (42). In mouse melanoma models, lactic acid was also shown to diminish interferon-gamma (IFN-γ) production in T cells, and increased lactate dehydrogenase expression was negatively correlated with T-cell activation (43). The secretion of lactate by tumor cells also affects the gradient across lactate transporters, which can blunt T-cell activation by diminishing their ability to recycle glycolytic byproducts (44, 45). Although lactate demonstrably inhibits the antitumor activity of effector T cells, it also promotes the development of immunosuppressive T_reg_ populations (46–49). Therefore, lactate accumulation in the tumor has a two-pronged immunosuppressive effect: it inhibits the activity of cytotoxic T cells and promotes the activity of T_reg_ populations.

The buildup of lactic acid in the TME is accompanied by a decrease in intratumoral pH, which also inhibits antitumor T-cell activity. As noted above, the acidity of the TME has been shown to decrease cytokine production by T cells and to increase T_reg_ subpopulations (25). In one study, the acidity of the TME was shown to inhibit CTL activity by completely blocking cytokine production and partially blocking lytic granule exocytosis; however, the functional activity of CTLs was recovered when the extracellular pH was neutralized (50). Separately, CD8+ T cells cultured at pH values that correspond to intratumoral pH (pH 6-6.5) exhibited an anergic state with reduced cytokine production and lower expression of CD25 and T-cell receptors (TCRs) (51, 52). Because the acidity of the TME is a major contributor to immune escape, several novel therapeutic strategies have focused on modulating TME pH. For instance, administering sodium bicarbonate has led to improved T-cell infiltration and increased responsiveness to immunotherapy in several murine models (53). Also, T cells genetically modified to overexpress *Hvcn1*, encoding for a voltage-gated H+ channel that selectively extrudes protons, demonstrated markedly increased antitumor functions (52). Collectively, lactic acid and the increased acidity of the TME are increasingly attractive targets for enhancing antitumor T-cell activity and bolstering immunotherapeutic strategies.

#### 2.1.2 Glucose depletion

The metabolic competition for glucose, among several other key metabolites, between tumors and T cells is a critical mediator of their relationship (27, 39). Tumor cells have been shown to outcompete T cells for glucose, which has detrimental effects on the cytolytic and effector functions of TILs (29). Mechanistically, restricted glucose consumption by T cells in a mouse sarcoma model led to reduced activity of the mammalian target of rapamycin (mTOR), which in turn led to decreased IFN-γ production and glycolytic capacity (39). Moreover, depletion of glucose in the TME not only reduces glycolytic flux in TILs but also decreases the generation of phosphoenolpyruvate, which has a critical role in sustaining TCR-mediated Ca^2+^-NFAT [nuclear factor of activated T cells] signaling; consequently, reduced production of phosphoenolpyruvate can markedly impair antitumor T-cell activity (54). Notably, depleted glucose levels in the TME can also provide a metabolic advantage to T_regs_ over CTLs, as T_regs_ are more reliant on fatty acid and lactate metabolism (55).

Apart from tumor-specific glucose depletion, limited glucose conditions have been shown to impair T-cell activity in several studies (56–58). Fundamentally, CTLs depend on aerobic glycolysis to supply energy for a vast array of activities and functions; therefore, it is unsurprising that glucose depletion in the TME hinders their cytolytic and effector functions. Notably, the adenosine monophosphate–activated protein kinase (AMPK) pathway has a central role in regulating glycolytic flux in both T cells and tumors. In T cells, activation of AMPK signaling promotes the proliferation of various cellular subsets (59). However, the AMPK pathway also has pleiotropic effects on cancer cells (13). Some studies report that its activation can help cancer cells survive metabolic stress, whereas others indicate that its activation can negatively regulate aerobic glycolysis, suppressing tumor growth (60, 61). As a nexus for T-cell and tumor glucose metabolism, the AMPK pathway is a key energy-sensing system that has been targeted with drugs such as metformin to enhance memory T cells (62). However, because metformin activates the AMPK pathway more globally, it may also promote T_reg_ expansion, consequently driving immunosuppression (13, 63). Nonetheless, it is important to recognize that the glucose-depleted conditions of the TME could be leveraged to develop T-cell priming strategies that improve their antitumor activity. One study reported that depriving T cells of glucose *in vitro* and inhibiting glycolytic flux improved CD8+ T-cell memory and antitumor activity (62). Transient glucose restriction was also shown to enhance CD8+ effector T-cell function and antitumor activity in mouse tumor models (64). Further research into the mechanisms underlying T-cell metabolic conditioning would provide greater insight into the bioenergetic plasticity of T cells.

#### 2.1.3 Reduced oxygen availability

The increased glycolytic flux in tumors, coupled with a disorganized vasculature, creates a hypoxic microenvironment that is not conducive to proper T-cell functioning (3). One of the major consequences of the hypoxic TME is T-cell exhaustion; the limited oxygen availability in the TME interferes with mitochondrial dynamics that coordinate TCR functioning and PD1 signaling (3, 65). Indeed, microenvironmental changes can alter pathways that sense oxygen tension, which is a key driver of T-cell exhaustion on a molecular basis (66). Hypoxia can also mediate immunosuppression by driving the recruitment of T_regs_ via CC-chemokine ligand 28 (CCL28) signaling, which promotes tolerance and angiogenesis (67). Upregulation of the transcription factor hypoxia-inducible factor 1-alpha (HIF-1α) in response to low oxygen conditions is also understood to increase glycolytic flux for cancer cells (68). Although the transcriptional activity of HIF-1α is critical for supporting tumor growth, its contribution to the hypoxic TME is also detrimental to effector T-cell function. For example, T cells have been shown to detect hypoxic conditions through oxygen-sensing prolyl-hydroxylase proteins, which subsequently limited T_H_1 responses, promoted induction of CD4+ T cells into T_regs_, and curtailed CD8+ T-cell effector functions in lung tumor models (69). Separately, HIF-1α was reported to upregulate PDL1 expression in cancer cells, facilitating escape from CTL-mediated lysis and promoting CTL apoptosis (70). Finally, tumor hypoxia was shown to promote the accumulation of extracellular adenosine (which inhibits effector T-cell activity) via HIF-1α-induced expression of the ATP-hydrolyzing ectonucleotidases CD39 and CD73 (71–73).

### 2.2 Fatty acid oxidation

The role of fatty acid oxidation (FAO) in modulating the activity of CD8+ TILs is controversial, with some studies suggesting that upregulated FAO supplies energy to sustain tumor-killing activity, and others that lipid accumulation in the TME drives T-cell exhaustion (74–76). As is true for glucose metabolism, T cells use FAO differently depending on their phenotype and activation status. For example, memory T cells upregulate fatty acid catabolism to maintain persistence and quiescence, whereas effector T cells rely on fatty acid anabolism to meet their demands for biomolecular mass (77, 78). In the TME, cancer cells have been found to exploit multiple sources of fatty acids. One of the most common mechanisms used to obtain fatty acids is *de novo* lipogenesis, facilitated by the upregulation of fatty acid synthase (79–81). However, cancer cells may also enhance uptake of lipids from the extracellular environment *via* receptor-mediated endocytosis of low-density lipoproteins, fatty acid-binding proteins, and/or fatty acid translocases (82, 83). Although altered fatty acid metabolism is an established feature of both T cells and cancer cells, it is important to contextualize how this metabolic change specifically informs the activity of T cells in the TME.

In the glucose-depleted conditions of the TME, CD8+ TILs often rely on fatty acids as an alternative energy source to fuel their antitumor activity; in mouse melanoma models, they were observed to increase fatty acid catabolism via peroxisome proliferator-activated receptor (PPAR)-α signaling (75). Further, because the early glycolytic activity of T cells is inhibited by PD1 signaling in the TME, T cells can switch to FAO to promote their longevity via increased expression of carnitine palmitoyltransferase 1A (CPT1A) (84). However, CD36-mediated uptake of fatty acids has also been shown to impair effector functions of CD8+ TILs by increasing ferroptosis and reducing the expression of pro-inflammatory cytokines (such as IFNγ and tumor necrosis factor [TNF]) (74, 85, 86). Moreover, the accumulation of long-chain fatty acids in the TME has been reported to blunt T-cell activity by reducing FAO (via downregulation of very-long-chain acyl-CoA dehydrogenase) and diminishing mitochondrial functions (76).

Apart from effector T cells, fatty acid metabolism also has a central role in supporting the activity of memory T cells. For instance, mice with T-cell–specific deletion of TNF receptor-associated factor 6 (TRAF6), a protein that modulates FAO, displayed robust CD8+ effector T-cell function, but they had profound defects in the ability to generate memory T cells (87). In the context of cancer, forced expression of proliferator-activated receptor gamma coactivator 1-alpha (PGC-1α), a protein that induces transcription factors that promote FAO and mitochondrial biogenesis, was found to improve CD8 T-cell central memory (88). PDL1 blockade in both *in vivo* and *in vitro* gastric adenocarcinoma models also increased fatty acid binding protein 4/5 (Fabp4/5) expression in resident memory T cells, improving their survival (89). Notably, the metabolism of T_regs_ is also characterized by an increased reliance on fatty acids for energy, which is advantageous for their survival in the glucose-depleted TME (90). FAO has also been linked with supporting the differentiation of CD4+ T cells into T_regs_, which contributes to the inhibition of antitumor immunity by increasing T_regs_ in the TME (91).

Although fatty acid metabolism is central to the interplay between T cells and tumors, its different roles in supporting or inhibiting the activities of various T-cell subsets (e.g., effector CD8+ T cells, memory T cells, exhausted T cells, and T_regs_) complicate its potential use as a therapeutic target. Nevertheless, several therapeutic strategies tested in preclinical tumor models have shown promising early findings (74). For instance, bezafibrate (an agonist of PGC-1α/PPAR complexes) increased FAO and the mitochondrial respiratory capacity of CTLs, supporting their antitumor immune function when used in conjunction with anti-PD1 therapy in mouse tumor models (92). In addition, genetic ablation of CD36 in T_regs_ not only decreased intratumoral T_reg_ populations but was also found to enhance the antitumor activities of TILs, especially when coupled with anti-PD1 therapy (90). Finally, inhibition of MEK1/2 was shown to enhance mitochondrial biogenesis and FAO in stem cell-like memory CD8+ T cells, improving their persistence and supporting antitumor activity in tumor-bearing mice (93). Although the overall dynamics of FAO in the context of TIL activity remain controversial, future research that aims to modulate FAO in combination with immunotherapies could reveal new avenues for selectively improving the activity of CD8+ TILs to create targeted antitumor treatment strategies.

### 2.3 Amino acid catabolism

Like glucose and fatty acids, amino acids are also subject to metabolic competition between cancer cells and T cells in the TME. Notably, cancer cells can rely on various endogenous and exogenous sources of amino acids to fulfill their bioenergetic demands. As one example, cancer cells have been shown to utilize *de novo* and/or salvage pathways of amino acid synthesis to produce serine (via phosphoglycerate dehydrogenase) (94). Tumors also depend on exogenous sources of both essential and non-essential amino acids to support their growth, and several amino acid transporters (e.g., SLC7A5 and SLC6A14) have been implicated in facilitating amino acid uptake for cancer cells (95, 96). Furthermore, cancer cells can synthesize amino acids from irregular sources by using transaminases that interconvert amino acids. For example, the aspartate transaminase and glutamic oxaloacetic transaminase were both found to critically support redox balance and growth in human pancreatic ductal adenocarcinoma cells (97, 98). Altogether, the multiple sources of amino acids that cancer cells can exploit to increase biosynthesis elucidate the broader pattern of tumors evolving to optimize overall nutrient consumption. For T cells, amino acids have crucial roles in supporting their activation and proliferation, as demonstrated by the upregulation of various amino acid transporters after activation (99). Glutamine is a particularly important catabolic substrate for activated T cells, as it provides a host of intermediate molecules that enter biosynthetic and mitochondrial metabolic pathways (100). Similarly, tryptophan and arginine have been shown to support T-cell proliferation, cytokine production, and activation marker expression (12, 101–103). Cancer cells not only compete with T cells for these crucial amino acids, but they also evolved mechanisms to degrade or remove them to facilitate immune escape. Several studies have indicated that tumors express tryptophan-degrading enzymes that hinder antitumor immunity by reducing the intratumoral accumulation of T cells (104, 105). These enzymes (particularly indoleamine 2,3-dioxygenase [IDO]) have emerged as novel targets that can be inhibited to bolster T-cell infiltration and activity in tumors. Mechanistically, depleted tryptophan in T cells via IDO activates the stress enzyme GCN2 kinase, which is stimulated when tRNAs are not charged with amino acids. Elevated GCN2 kinase prevents T-cell proliferation and causes anergy (106). In orthotopic and metastatic models of liver cancer, delivery of shRNA for IDO resulted in the upregulation of T_H_1 cytokines (interleukin [IL] -12 and IFNγ) as a part of improved CD8+ T-cell cytotoxic activity (107). Administration of the receptor tyrosine kinase inhibitor imatinib was shown to downregulate the expression of IDO, resulting in activation of CD8+ T cells and induction of apoptosis of T_regs_ in mouse models of gastrointestinal stromal tumors (108). Finally, selective inhibition of IDO with the small molecule INCB024360 was found to improve T-cell proliferation and promote IFNγ production, thereby inhibiting tumor growth in a lymphocyte-dependent manner (109). In summary, several types of tumors demonstrably rely on IDO to outcompete T cells for tryptophan, but the emergence of several novel therapeutics that inhibit IDO lend significance to further efforts to combine IDO inhibitors with immunotherapy to strengthen antitumor T-cell activity.

The depletion of glutamine in the TME has similarly profound inhibitory consequences for TILs. However, the overall role of glutamine in mediating T-cell activity, particularly within the TME, is highly complex. T cells utilize glutaminase to convert glutamine into glutamate, supporting protein synthesis, redox balance, and the TCA cycle. However, glutaminase differentially affects the activity and differentiation of helper T-cell subsets via altered chromatin accessibility. Glutaminase deficiency was shown to initially reduce T-cell activation/proliferation and impair T_H_17 differentiation, but later promoted the effector function of CD4+ T_H_1 cells and effector CD8+ T cells (110). In the context of the TME, glutaminase inhibition contrarily impaired CD8+ T-cell activation in *STK11-/Lkb1-*deficient models of lung adenocarcinoma. *Lkb1-*deficient tumors were shown to have significantly increased glutamine production, suggesting that immunotherapy could be combined with glutamine inhibition to support glutamine availability for CD8+ T cells. However, the authors of this study demonstrated that inhibiting the PD1/PDL1 axis (using immunotherapy) as well as glutaminase activity in T cells in the TME reduced effector differentiation and cytotoxicity (111). A separate study comprehensively inhibited glutamine metabolism, which intriguingly resulted in potent anti-tumor effects but preserved effector T-cell activity. Tumor-bearing mice treated with comprehensive glutamine blockade demonstrated reduced hypoxia, acidosis, and nutrient depletion. On the other hand, glutamine metabolism blockade enhanced the function of anti-tumor immune cells by conditioning TILs toward a long-lived, memory-like phenotype that displayed high proliferation, activity, and effector functioning (112). Apart from T cells, suppressive myeloid cells in the TME can also be targeted *via* glutamine metabolism blockade. One investigation demonstrated that glutamine metabolism blockade with 6-diazo-5-oxo-L-norleucine (DON) inhibited the generation and recruitment of myeloid-derived suppressor cells (MDSCs). The study also reported that inhibiting glutamine metabolism could reduce immunosuppressive effects in the TME by inhibiting IDO expression and decreasing kynurenine levels, which could indirectly support T cell activation (113).

Glutamine starvation was found to blunt T-cell proliferation and cytokine production, which is consistent with previous findings affirming the importance of glutamine import in supporting T-cell activation (114). Glutamine restriction also impaired the effector activities of CD8+ T cells, but *ex vivo* culture under glutamine-restricted conditions followed by reinfusion led to extended survival in tumor-inoculated mice (115). Glutamine restriction has also been shown to reduce nucleotide biosynthesis in T cells, promoting the development of T cells with high FOXP3 expression and regulatory properties (116). Several therapeutic strategies centering on glutamine metabolism have been tested such as glutamine transport inhibitors, which prompted reduced tumor growth in lung and prostate cancers (13, 117, 118). Nonetheless, further research is needed to elucidate the dynamics between glutamine uptake in the TME and its consequences for antitumor T-cell activity. A stronger understanding of these dynamics could inform the development of combination therapies that more selectively kill cancer cells while sustaining T-cell function.

Arginine is also critical in modulating T-cell metabolism by enhancing their survival and antitumor functions (119). The expression of arginase by MDSCs in the TME can deplete intratumor L-arginine, resulting in T-cell anergy and cell-cycle arrest of T cells in the G0-G1 phase (120). Moreover, increased expression of arginase by MDSCs in patients with renal cell carcinoma was correlated with decreased cytokine production and lower expression levels of the TCR CD3-ζ chain (121). In organ cultures of human prostate carcinomas, inhibiting the activity of arginase was found to restore TIL responsiveness to tumors by polarizing their cytotoxic granules (122). Aside from arginase, nitric oxide synthase is also responsible for metabolizing arginine. The administration of aspirin, which releases nitric oxide and inhibits nitric oxide synthase in MDSCs, was shown to increase the number and function of tumor-antigen–specific T lymphocytes against primary mammary carcinoma cell lines (123). L-arginine is another crucial metabolic target being explored in several clinical studies with the goal of developing more effective immunotherapies (124).

Branched chain amino acids (BCAAs) also play critical roles in supporting the activity of both cancer cells and T cells. BCAAs, especially leucine, have been shown to promote mTORC1 activity in T-cells, which regulates T-cell differentiation and function (125, 126). Furthermore, the import of BCAAs requires the L-type amino acid transporter (LAT). One member of the LAT family, LAT1, plays an especially important role in facilitating the increased uptake of BCAAs during T-cell activation (127). Two notable branched chain amino transferases, BCATc and BCATm, are responsible for catalyzing the first step in the degradation of the BCAAs isoleucine, leucine, and valine (128). Although several studies have shown that BCAT isoenzymes are highly expressed across different cancer types, the importance of these enzymes in mediating immunosuppressive effects is only recently garnering attention (128–130). One investigation reported that *BCAT1* expression was positively correlated with immune checkpoint genes in several cancers (131). Another study demonstrated that BCAT1 was correlated with immunosuppressive status in IDH1 wild-type gliomas (132). The underlying mechanism that explains how BCATs in the TME specifically modulate T-cell activity has not yet been clarified, but early findings have already identified these amino transferases as potential pharmaceutical targets.

## 3 Tumor metabolites in T-cell dysfunction

The TME is a dynamic and complex environment consisting of resident cells, infiltrating cells, secreted signaling factors, and an extracellular matrix. Tumors depend on the TME for survival and growth; they can remodel the microenvironment by promoting angiogenesis and immune evasion via extracellular factors collectively classified as hallmarks of cancer (1). However, in addition to inducing angiogenesis, increased metabolic activity and the highly proliferative nature of cancer cells cause the depletion of key nutrients in the microenvironment. The hypoxic, acidic, and nutrient-depleted conditions in the TME are not hospitable for immune cells (133–135). Moreover, the reprogramming of cancer cell metabolism allows those cells to adapt to harsh conditions and dampens antitumor immunity through metabolic immune checkpoints (5, 135, 136). The various metabolic pathways exploited by tumors result in either consumption or production of key metabolites that have a variety of salient regulatory effects on T-cell activity (**Table 1**).

**Table 1:** Effects of select tumor microenvironment metabolites on T-cell activity.

Metabolite	Relationship to Tumor	Effect on T Cells	References
Glucose	Consumed via aerobic glycolysis	Reduced effector functions Decreased activation Increased exhaustion and PD1 signaling	(29, 137–141)
Lactate	Produced via aerobic glycolysis	Reduced proliferation Increased apoptosis induction Decreased cytokine production	(15, 40–45)
Adenosine	Produced via ATP metabolism	Blunted antitumor functions Decreased cytokine production Increased PD1 signaling Reduced proliferation	(142–146)
Kynurenine	Produced via tryptophan decomposition	Increased PD1 signaling Reduced proliferation Increased exhaustion/immunosuppression	(147–149)
Cholesterol	Enriched in TME via exogenous uptake or *de novo* synthesis	Reduced effector functions Increased endoplasmic reticulum stress Decreased cytokine production	(150, 151)
Glutamine	Consumed via rapid glutaminolysis	Reduced proliferation Decreased cytokine production Reduced effector functions	(114, 115)
Arginine	Consumed via rapid uptake and enzyme-mediated (arginase) degradation	Reduced survival Blunted antitumor functions Anergy and cell-cycle arrest Decreased cytokine production	(119–122)
Tryptophan	Consumed via rapid uptake and enzyme-mediated (IDO) degradation	Reduced effector functions Decreased activation Reduced proliferation Decreased cytokine production	(104–109)
ROS	Produced via several metabolic, enzymatic, and dysfunctional processes	Increased apoptosis Increased exhaustion Reduced effector functions	(152–154)
Ammonia	Produced via dysregulated urea cycle	Increased exhaustion Reduced proliferation Reduced effector functions	(155)
Polyamines	Enriched in TME via increased uptake and synthesis	Increased immunosuppression	(156–158)

ATP, adenosine triphosphate; TME, tumor microenvironment; IDO, indoleamine 2,3-dioxygenase.

### 3.1 Glucose

Cancer cells are well known to increase their glucose uptake and switch from oxidative phosphorylation to aerobic glycolysis. On a clinical level, overexpression of the glucose transporter GLUT1 in cancer cells has been linked with poor prognosis in several types of solid tumors (159). Increased glucose uptake and glycolytic flux by cancer cells limit glucose utilization by T cells and resulted in low T-cell infiltration and impaired intratumoral activity (137, 138). Another study also provided evidence of restricted glucose consumption among T cells in models of ovarian cancer. In that study, the reduction in glucose consumption constrained the expression of the methyltransferase EZH2 in T cells via specific microRNAs. EZH2 is responsible for epigenetic induction of Notch signaling by repressing Notch inhibitors; activated Notch signaling regulates the polyfunctionality and survival of T cells (139). Restricting glucose utilization by T cells also contributes to immune exhaustion, especially because glucose consumption is a limiting factor for T-cell activation. The costimulatory molecule CD28 is required for activation after TCR stimulation, and CD28 increases glycolytic flux and glucose consumption via GLUT1 expression and cell surface trafficking by Akt-dependent and -independent pathways in response to activation (27, 140). PD1 expression similarly affects T-cell glucose metabolism, and reduced glucose levels enhance PD1 expression in T cells (39, 84). Further, restricting glucose increases the susceptibility of T cells to apoptosis via Bcl2 proteins (141). Collectively, these results indicate that glucose is a critical metabolite for sustaining T-cell activity and antitumor T-cell activity, but its rapid consumption by cancer cells can dampen T-cell activity through a variety of immunosuppressive mechanisms.

### 3.2 Lactic acid

As previously discussed, increased aerobic glycolysis also results in lactic acid accumulation in and acidification of the microenvironment. Increased amounts of the enzyme lactic acid dehydrogenase have been linked with poor outcomes for patients with various types of cancer (150, 160–163). Notably, excessive lactic acid in the TME affects cancer cells and immune cells differently. Lactic acid accumulation inhibits the proliferation, cytokine production, and chemotaxis of natural killer (NK) cells and T cells and promotes apoptosis of these cells (15, 43). Importantly, the abundance of lactic acid in the TME has profound implications for the activity of tumor associated macrophages (TAMs) in addition to T-cells. Macrophages exhibit phenotypic plasticity and can differentiate into different polarized states depending on specific microenvironmental cues (164). M1 macrophages are largely responsible for mediating pro-inflammatory and anti-tumor responses, whereas M2 macrophages predominantly mediate anti-inflammatory and pro-tumor immune responses (165). TAMs can be polarized into either of these subtypes in response to the various metabolites and signaling molecules present within the TME. For instance, increased lactic acid levels in the TME enhance the polarization of pro-tumorigenic M2 macrophages via the ERK/STAT3 signaling pathway in breast cancer models (166). The acidic environment created by tumor cells, independent of lactate, also contributes to M2 macrophage polarization in the TME in prostate cancer models (167). Lactic acid-induced acidosis also suppresses the differentiation and maturation of dendritic cells; the resulting reduction in antigen presentation to T cells negatively affects adaptive immunity (168, 169). In addition, lactic acid was shown to enhance the activity of the immunosuppressive MDSC population via the HIF-1α pathway as well as disrupting neutrophil antitumor function (170, 171). Separately, lactic acid promotes angiogenesis via the HIF-1α/VEGF axis in the tumor microenvironment, contributing to tumor growth and the development of distant metastases (172). Despite growing evidence implicating lactate as an immunosuppressive metabolite, it is important to recognize that the broader role of lactic acid in T-cell biology is complex (173). On one hand, lactate can support tricarboxylic acid (TCA) cycle anaplerosis in effector T cells by serving as a physiologic carbon source (48). One study reported that lactate is a mitochondrial fuel for CD8+ effector T cells that contributes to TCA metabolism, and when it is readily available, effector T cells will preferentially oxidize lactate to support overall viability (174). On the other hand, the immunosuppressive effects of lactic acid accumulation may be limited to the hypoxic conditions of the TME, making it a promising target for cancer treatment strategies (173). Preclinical findings indicate that blocking lactic acid dehydrogenase limits tumor growth and improves immune checkpoint therapy (175, 176). Another approach involves blocking the MCT4 or MCT1 receptors, which are responsible for transporting lactic acid from inside the cell to the microenvironment. Targeting these receptors can improve T-cell function and immunogenicity by blocking lactic acid accumulation and acidification (177, 178).

### 3.3 Adenosine

Increased inflammation in the TME enhances the metabolism of extracellular ATP or adenosine diphosphate (ADP) to adenosine monophosphate (AMP). AMP is subsequently converted into adenosine by the ectonucleotidases CD39 and CD73, which are highly expressed on stromal cells, tumor cells, immune cells, and endothelial cells. Although extracellular ATP is immunogenic, accumulation of the adenosine metabolite in the TME has inhibitory effects on the immune system. For example, adenosine signaling via A2a receptors, which are G-coupled receptors on immune cells, was shown to dampen the antitumor response (142, 143). Increased protein kinase A activity, because of downstream of A2a receptor signaling, also has suppressive effects on effector T-cell function by reducing cytokine production and cell proliferation. Moreover, A2a receptor signaling increases T_reg_ differentiation and enhances the immunosuppressive activity of T_regs_ (144). Notably, adenosine also enhances the expression of other immune checkpoints, including CTLA4, PD1, and LAG3 (145, 146). The benefits of targeting the adenosine axis and preventing adenosine accumulation in the TME have been extensively studied in preclinical models. For example, in murine models, blocking the adenosine axis was found to reduce tumor growth and prevent the formation of distant metastases (144, 179, 180). Blocking the adenosine axis also increased CD8+ T-cell infiltration into tumors, enhanced inflammatory cytokine production, and synergized effectively with other immune checkpoint therapies (181–184).

### 3.4 Cholesterol

Evidence is growing on the importance of cholesterol in supporting tumor progression, and altered cholesterol biosynthesis programs are increasingly recognized as important features of various cancers, including melanomas and sarcomas (185). Indeed, upregulated *de novo* biosynthesis of cholesterol, coupled with increased exogenous uptake, are consistently important in facilitating tumor growth and survival (186). On an immunologic basis, accumulation of cholesterol in the TME can induce metabolic and endoplasmic reticulum stress in T cells, resulting in exhaustion and reduced effector functions, including cytokine production (150). Further, a positive correlation was found between tumors enriched in cholesterol and CD8+ T cells with upregulated expression of immunosuppressive molecules such as PD1, TIM3, and LAG3 (150). Separately, inhibition of cholesterol esterification via genetic ablation or pharmacologic inhibition of ACAT1 (a cholesterol esterification enzyme) was found to potentiate an antitumor immune response for CD8+ T cells. These investigators showed that ACAT1-deficient CD8+ T cells were better than wild-type CD8+ T cells at suppressing tumor growth in mouse melanoma models (151).

### 3.5 ROS and ammonia

Increased inflammation within the TME also results in the accumulation of reactive oxygen species (ROS), which can form from a variety of processes including mitochondrial dysfunction, oncogene activity, and increased oxidase activity (187). Excessive ROS and oxidative stress cause field cancerization and metastasis by promoting oncogenic pathways that prevent cancer cell apoptosis (188). Further, ROS affect M2 macrophage polarization in ways that induce T-cell apoptosis (152). Tumor-induced MDSCs promote cancer progression and suppress antitumor immunity via ROS (153). In addition, under hypoxic conditions, T-cell activity is disrupted via abnormal mitochondrial function that creates mitochondrial ROS that increase exhaustion through reduced PGC-1α gene expression, which is responsible for mitochondrial biogenesis (154). Antioxidant inflammation modulators are used to target ROS that bind Keap1 and prevent degradation of the nuclear factor erythroid 2–related factor Nrf2. Increased Nrf2 diminishes ROS levels and inflammation in the TME, consequently controlling tumor growth and metastasis (189).

Accumulation of high levels of ammonia in the microenvironment resulting from disruption in the urea cycle also blunts antitumor immunity by exhausting T cells and reducing their proliferation. In patients with colorectal cancer, increased serum ammonia and ammonia-related genes are associated with poor outcomes and immune checkpoint resistance. In mouse models, enhancing the clearance of tumor-associated ammonia rescued T-cell function and improved the efficacy of anti-PD1 immunotherapy (155).

### 3.6 Kynurenine and polyamines

Kynurenine is another immunosuppressive metabolite produced via tryptophan catabolism, and that IDO enzyme-catalyzed reaction is a rate-limiting step in the kynurenine pathway. Kynurenine is related to aging, inflammation, and modulation of ROS levels via aryl hydrocarbon receptors (190). Kynurenine also increases IDO expression in tumor cells through an autocrine aryl hydrocarbon receptors–IL-6–STAT-3 loop (191). Further, elevated kynurenine levels cause exhaustion and increased expression of immune checkpoint markers by tumor-infiltrating CD8+ T cells. Targeting kynurenine via IDO blockade has synergized with immune checkpoint therapy and improved antitumor immunity in preclinical and clinical studies (147–149).

Polyamines (e.g., putrescine, spermine, spermidine) are aliphatic cationic metabolites that are synthesized from arginine and glutamine and have essential functions in the proliferation of normal and neoplastic cells (192). At physiological concentrations, they also contribute to T-cell activation and differentiation (193). On the other hand, cancer cells show increased polyamine synthesis and uptake. The accumulation of polyamines in the TME produces an intricate immunosuppressive effect (156, 157). Targeting polyamines has been explored in the context of cancer immunotherapy; dietary polyamine deprivation has been shown to deter tumor-induced immunosuppression by increasing IL-2 levels (158). In another study, polyamine-blocking therapy, combined with the inhibition of ornithine decarboxylase (the rate-limiting enzyme of polyamine synthesis), reversed immunosuppression in the TME by reducing MDSCs and increasing infiltrating T-cell populations (157).

### 3.7 Succinate

Tumors with loss-of-function mutations in succinate dehydrogenase have high levels of succinate present in the TME (194). While the effects of succinate accumulation on innate immune cell activity have been investigated, few studies have explored the role of succinate in modulating T-cell activity within the TME. A key study interrogating this relationship showed that high succinate levels in the TME can inhibit CD4+ and CD8+ T-cell effector function, particularly by blunting cytokine secretion (IFN-γ) and degranulation (195). Mechanistically, increased succinate uptake was found to inhibit T-cell succinyl-CoA synthetase activity, glycolytic flux, and the TCA cycle. These effects cumulatively suppressed T-cell activity, but restoring TCA cycle flux was shown to reverse the succinate-mediated suppression (195).

## 4 Metabolism-mediated epigenetic modulation of T-cell activity in tumors

The metabolic reprogramming of T cells within tumors is inextricably linked to epigenetic modifications, and a growing body of evidence suggests that the metabolism-epigenetics relationship in T cells could also be a therapeutic target for cancer (196). Generally, the epigenetic regulation of certain genes in T cells is understood to be critical for supporting their proliferation, activity, and native functioning. For instance, histone methylation and the corresponding activity of demethylases (e.g., KDM6B) has been shown to support the virus-specific CD8+ T-cell response as well as CTL effector programs (85). Similarly, histone acetylation—namely, acetylation of histone H3 lysine 9 (H3K9)—has also been reported to facilitate a robust CD8+ memory T-cell response by upregulating the expression of various effector molecules, including perforins and granzyme B (197). Although histone acetylation and methylation are among the most widely studied epigenetic modifications that T cells undergo during activation, evidence is accumulating to suggest that certain metabolites can also epigenetically regulate T-cell activity. As one example, β-hydroxybutyrate, a metabolite in the ketogenic pathway, can epigenetically modify H3K9, supporting CD8+ memory T-cell development (198). Separately, there is a growing body of evidence broadly supporting the role of epigenetics in regulating T-cell exhaustion. In fact, exhausted T cells have been characterized by a distinct T-cell chromatin state and shared differentiation program regulated by specific transcription factors (199). One study even reported that transcriptional and epigenetic mechanisms are responsible for a four-stage hierarchical developmental pathway that results in CD8+ T-cell exhaustion in subsets defined by Ly108 and CD69 (200). As the role of epigenetics in regulating T-cell phenotype and activity is further clarified, the dynamic intersection between epigenetics and metabolism is increasingly relevant for guiding therapeutic interventions. Here, we briefly summarize the roles of three epigenetic regulatory mechanisms—acetylation, methylation, and metabolite modulation—in mediating T-cell metabolism, specifically in the context of cancer.

### 4.1 Acetylation

In the glucose-depleted conditions of the TME, T cells can utilize acetate as an alternative energy source to support growth and effector functions (201). As with the aforementioned metabolites, acetate can also be obtained by cancer cells through exogenous or endogenous sources. Acetate in blood plasma, which may be derived from the diet or even the gut microbiome, can be taken up by cancer cells through various transport proteins, including monocarboxylate transporter-1 (202). The nucleocytosolic acetyl-CoA synthetase enzyme, ACSS2, has also been reported to play a key role in facilitating acetate uptake across various human tumors (203). Notably, the findings from this study also suggest that ACSS2 may be responsible for converting acetate released from deacetylated proteins into an acetyl-CoA pool that can be used for epigenetic regulation (203, 204).

Besides serving as a substrate for energy production, acetate can also promote histone acetylation and chromatin accessibility in T cells, enhancing IFN-γ gene transcription and cytokine production (205). Separately, the inhibition of mitochondrial pyruvate carrier (MPC) has been shown to promote acetyl-CoA production, which enhanced histone acetylation and the transcription of pro-memory genes (such as *Sell, Tcf7*, and *Ccr7*) that are critical for CD8+ memory T-cell function (206). However, that same study found that ablation of MPC blunted the antitumor function of CD8+ T cells, because MPC sustains lactate oxidation for T cells in the TME. Nevertheless, this antitumor activity could be circumvented by using a small-molecule MPC inhibitor to imprint a memory phenotype during chimeric antigen receptor-T cell (CAR-T) expansion. CAR-T cells are engineered to express synthetic receptors that can recognize specific antigens expressed by cancer cells and potentiate a powerful anti-tumor response (207). However, certain limitations (such as exhaustion and senescence) of CAR-T therapy have brought attention to conditioning strategies that could improve the potency and persistence of CAR-T cells (208). Indeed, the conditioning strategy involving an MPC inhibitor resulted in superior CAR-T antitumor activity upon adoptive cell transfer immunotherapy. These early findings indicate that histone acetylation, particularly acetylation mediated by acetate or acetyl-CoA, could be strategically targeted alongside various metabolic pathways to bolster antitumor T-cell activity (196).

### 4.2 Methylation

In the hypoxic TME, HIF-1α and von Hippel-Lindau (VHL) proteins are essential for regulating CD8+ T-cell metabolism and function. The VHL-HIF-1α axis (in response to hypoxic conditions) and TCR activation have been shown to upregulate the production of 2-hydroxyglutarate, a molecule that can epigenetically modulate T-cell phenotype and activity, but in contradictory ways (196, 209). One group found that 2-hydroxyglutarate, an oncometabolite, induced global changes in histone H3 methylation by inhibiting H3K27me2/3 demethylase, which promoted CD8+ T-cell lymphocyte proliferation, persistence, and antitumor capacity (209). However, another study reported that the overproduction of 2-hydroxyglutarate in the TME (due to gain-of-function mutations in isocitrate dehydrogenase) decreased CD8+ T-cell cytotoxicity and impaired IFN-γ production, albeit not through epigenetic reprogramming of T-cell metabolism (210). Instead, that study found that 2-hydroxyglutarate inhibited lactate dehydrogenase activity, which decreased the regeneration of cytosolic NAD+ that is ordinarily needed to support T-cell glycolysis.

Tumor cells can also upregulate the methionine transporter SLC43A2 to rapidly take up methionine and prevent T cells from metabolizing it. Reducing intracellular methionine in CD8+ T cells resulted in the loss of demethylation at H3K79me2, causing T-cell apoptosis and dysfunction. Inhibiting SLC43A2 recovered T-cell methionine metabolism and also improved checkpoint blockade antitumor immunity in preclinical models (211). A similar strategy involving metabolic reprogramming through altered methylation patterns was used to decrease the infiltration of T_regs_ in solid tumor models. Supplying α-ketoglutarate altered the methylation pattern of naïve CD4+ T cells activated under T_reg_ polarizing conditions, which led to reduced T_reg_ differentiation and decreased FOXP3 expression among adoptively transferred cells. These investigators suggested that α-ketoglutarate reprograms T cells to increase mitochondrial metabolism, which may affect the fate of CD4+ T-cell (specifically, T_reg_) differentiation (212). Further research into the intersection of DNA methylation patterns and T-cell metabolism is warranted to inform a stronger mechanistic understanding of how this intersection can be therapeutically harnessed..

### 4.3 Metabolites

T-cell gene expression can also be regulated epigenetically by select metabolites; however, research on this regulation in the context of cancer is still in early stages. The epigenetic effects of one such metabolite, lactate, are being investigated to clarify its role in modulating anti-cancer T-cell immunity. One recent study found that delivering sodium lactate, but not glucose, to tumor-bearing mice increased the stemness of CD8+ T cells, resulting in tumor growth inhibition. Mechanistically, the lactate inhibited histone deacetylase activity, increasing acetylation at H3K27 and expression of Tcf7, thereby supporting antitumor immunity (213). Arginine is another critical metabolite that can epigenetically influence T-cell metabolism to promote antitumor activity. Increased L-arginine levels have been shown to induce a metabolic shift from glycolysis to oxidative phosphorylation in T cells, promoting a central memory-like phenotype with increased survival capacity. These metabolically reprogrammed T cells had greater antitumor activity in mice bearing B16 melanoma tumors. Strikingly, these investigators also reported that L-arginine induced structural changes in three transcriptional regulators (BAZ1B, PSIP1, and TSN) that ultimately were responsible for mediating pro-survival effects in T cells (119). In short, the growing interest in metabolites and metabolic enzymes as epigenetic regulators of T-cell activity is opening new avenues for the development of more effective cancer immunotherapies. Nevertheless, further research into how specific metabolites regulate T-cell gene expression would be beneficial for informing precise strategies that can bolster antitumor immunity while circumventing T-cell exhaustion and/or dysfunction (196).

## 5 Molecular strategies for targeting metabolism to enhance antitumor T-cell activity

The framework of immunometabolism has revealed a new set of therapeutic targets that can be exploited to enhance immune responses. As previously stated, transcriptional regulation of differentiation, effector functions, and longevity of T cells are all fundamentally tied to cellular metabolism. The goal in immunometabolism is to explore and investigate ways to modulate the metabolic programs that govern these effects to manipulate and enhance immune responses against cancer. In this section, we review some of the metabolic pathways that have been explored in strategies to improve antitumor T-cell function.

One of the most salient metabolic targets is the PI3K/AKT/mTOR signaling pathway, which induces glycolysis downstream of T-cell activation. Several studies have shown that sustained activation of this pathway is correlated with terminal differentiation and loss of T-cell memory. An investigation of the AKT inhibitor A-443654 to increase the number of memory CD8+ T cells showed that AKT inhibition led to the transition of a fraction of short-lived effector CD8+ T cells to memory cells (214). Further research into AKT inhibition revealed that TILs that had been expanded with AKT inhibitors had significantly increased expression of the central memory phenotype marker CD62L. The evolutionarily conserved serine/threonine kinase mTOR has a central role in T-cell differentiation and function, as it mediates the induction of glycolysis upon T-cell activation, which is necessary for naïve CD8+ T cells to differentiate into effector CD8+ T cells (215). Administration of the mTOR inhibitor rapamycin, which induces FAO, led to a significant increase in memory CD8+ T-cell development and corresponding recall response (87).

Meanwhile, research on the effect of other mTOR inhibitors on effector T cells has shown mixed results. In one study, the addition of temsirolimus, a rapamycin analogue, enhanced the activation and function of effector T cells stimulated with a heat shock protein–based antitumor vaccine. Temsirolimus also enhanced memory CD8+ T-cell function, corroborating the findings obtained with rapamycin (216). In other studies, however, rapamycin was shown to have immunosuppressive effects that impeded lymphocyte recruitment to tumor-draining lymph nodes as well as recruitment of CD8+ T cells to the tumor (216). FAO is well known for its importance in the formation of memory CD8+ T cells, as evidenced by the deleterious effects on T-cell activity that arise from deficiencies of selected FAO enzymes. Mice with T-cell–specific deletion of TRAF6 exhibit changes in the expression of genes that regulate FAO and show profound defects in the ability to generate memory T cells (87). Conversely, enforced expression of FAO enzymes can yield favorable results.

Another metabolic pathway target is CPT1A, an enzyme essential for FAO. This target can be exploited through adoptive T-cell therapy, a technique in which a patient’s own T cells are isolated, cultured *in vitro*, and then transferred back to the patient. Adoptive transfer of OT-I T cells transduced *ex vivo* with CTP1a resulted in increased numbers of memory T cells compared with control transduced OT-I T cells in a mouse model (217). This same mechanism of adoptive cell transfer was used to examine the effect of pathway modulation in targeting glycolysis. Expression of the glycolytic enzyme Pgam1 induced CD8+ T cells to adopt a predominantly glycolytic phenotype, and adoptive transfer of Pgam1-transduced T cells showed decreased long-term survival in both lymphoid and peripheral tissue (62). That group also investigated whether inhibiting glycolysis with the H2K inhibitor 2DG would conversely enhance T-cell survival and the capacity for memory T-cell generation. The phosphorylated form of the glucose analog 2DG-6-P was found to accumulate within cells and to inhibit hexokinase activity. Adoptively transferred 2DG-treated pmel-1 CD8+ T cells were shown to expand robustly in wild-type mice infected with gp100-VV. Moreover, a higher proportion of 2DG-treated cells retained a memory phenotype, whereas most control CD8+ T cells underwent terminal differentiation (62).

The 2DG molecule can also be used in manufacturing CAR-T cells, which include genetically engineered self-CD8+ TILs that are expanded before being re-introduced into the patient. During expansion, the cells can be cultured with nutrients that support any metabolic challenges encountered when competing in the TME (218). Glycolysis inhibition, for example, is crucial for the manufacturing process of CAR-T cells to maintain an undifferentiated state that also persists for prolonged periods *in vivo*. This can take many forms, including optimizing the medium to enhance mitochondrial function and indirectly inhibiting glycolysis. As one example, the addition of arginine to the medium can promote oxidative phosphorylation and inhibit glycolysis (219). Many cytokines (e.g., IL-7, IL-15, IL-21) can also be added to the medium to shift metabolic programs away from glycolysis (9). Another way of enhancing mitochondrial metabolism in CAR-T cells is by using PGC-1α activators, as discussed earlier (88). Enhancing mitochondrial metabolism can also support memory T cells. CAR-T cells manipulated with the co-stimulatory activator 4-1BB displayed increased mitochondrial synthesis and FAO, supporting their antitumor activity (220). Finally, a CRISPR activation screen identified proline metabolism a potential target for enhancing CAR-T therapy (221). Specifically, the authors of the study identified *PRODH2*, encoding for proline dehydrogenase 2, as an ideal gain-of-function target that could be leveraged to enhance CAR-T therapy. Indeed, the targeted genomic knock-in or lentiviral overexpression of *PRODH2* boosted CAR-T killing of cognate cancer cells.

ICIs can also be used to manipulate T-cell metabolism. PD1 signaling inhibits T-cell activation by altering glucose uptake and glycolysis (222). CTLA4 signaling also impairs glycolysis during effector T-cell activation (84). PDL1 and B7H3 support malignant cells in the TME by enhancing aerobic glycolysis via activation of the HIF-1α and PI3K/AKT/mTOR pathways (223). Blocking these immune checkpoint inhibitors is therefore beneficial for T-cell activation, proliferation, and survival. CTLA4 blockade promotes cytotoxic CD8+ T-cell priming while inhibiting tumor-promoting T_regs_ (224). PD1 blockade can also aid in the differentiation of progenitor CD8+ T cells (222). Moreover, blocking PD1 inhibits the mTORC1 pathway, which leads to impaired glycolysis in the TME (39). Overall, the progression in our understanding of the metabolic mechanisms underlying T-cell activity will open new avenues for leveraging strategies such as adoptive cell transfer, CAR-T therapy, and ICIs to design more effective therapies.

Finally, apart from directly targeting cancer cells that metabolically modulate T-cell activity, stromal players in the TME can also be targeted due to their adverse effects on T-cell activity. For instance, the elimination of cancer-associated fibroblasts (CAFs) can facilitate the improved metabolic activity and cytotoxic effects of CD8+ T cells (225). One marker of CAFs, fibroblast activation protein–α (FAP), was targeted by combining a traditional cancer vaccine with a vaccine that selectively targets FAP+ fibroblasts. This strategy reduced tumor progression in mouse melanoma models by alleviating metabolic stress on CD8+ T-cells, potentially by increasing glucose availability (226). Furthermore, in prostate cancer models, the release of lactate by CAFs was found to reduce the proportion of anti-tumor T_H_1 susbsets and increase T_reg_ cells. Targeting the TLR8/miR21 axis, which is activated by this CAF-immunomodulated environment, could therefore be a viable therapeutic strategy (227). Stroma-associated pancreatic stellate cells can also modulate metabolic activity in pancreatic ductal adenocarcinoma by secreting alanine as a carbon source to fuel the TCA cycle in cancer cells. These pancreatic stellate cells can therefore provide a metabolic advantage to cancer cells over effector T-cells in pancreatic ductal adenocarcinoma (228). Altogether, the stromal cells of the TME can serve as important targets for therapeutic intervention (e.g., by vaccination or molecular inhibition) to bolster an anti-tumor immune response.

## 6 Clinical significance of targeting metabolism to improve immunotherapy

The advent of ICIs has revolutionized cancer immunotherapy. In less than a decade, significant improvements have also been observed in response rates, as the percentage of patients estimated to respond to checkpoint inhibitor drugs increased to from 0.14% in 2011 to 12.46% in 2018 (229). However, this response rate is still far from optimal, underscoring the need to either develop other treatment options or modify current approaches. As discussed throughout this review, cancer cells can suppress an antitumor response by depleting essential nutrients or reducing the metabolic fitness of tumor-infiltrating cells (230, 231). Therefore, tailoring immune responses by manipulating cellular metabolic pathways may improve clinical outcomes. Indeed, several clinical studies have either been conducted or are currently underway to examine combinations of metabolic targets and immune checkpoint inhibition to bolster antitumor immune responses.

As noted previously, T cells undergo metabolic reprogramming to fuel their bioenergetic needs based on their function. Naïve T cells depend largely on oxidative phosphorylation to survive (19). However, upon exposure to antigens, naïve T cells differentiate into effector cells that utilize amino acids (e.g., glutamine) and glucose for proliferation and cytolytic activity (232). If the antigen is cleared, the T cells differentiate into long-lived memory cells; however, if the T cells fail to clear the antigen, as is often the case in cancer, then the T cells become exhausted. Exhausted T cells generally exhibit dysfunctional mitochondria and decreased mitochondrial mass. Notably, they also exhibit gene signatures that have been correlated with poor prognosis in hepatocellular carcinoma patients, but also represent therapeutic targets (233). Moreover, inhibitory receptors like PD1 and CTLA4 also impair T-cell metabolism. For instance, T cells exposed to PD1 signaling showed decreased rates of glycolysis and glutaminolysis but increased rates of FAO (84, 234).

Metabolic reprogramming has emerged in recent years as a novel means of reversing T-cell exhaustion in the TME, with promising results obtained in both animal models and *in vitro* studies. However, whether metabolic reprogramming with ICIs has beneficial effects in humans remains unclear. To our knowledge, at least 15 clinical studies either have been completed or are currently underway to evaluate the effects of targeted T-cell metabolic inhibitors in conjunction with ICIs. Three of these studies have been withdrawn (NCT04231864) or terminated (NCT03894540, NCT04265534) for feasibility issues, lack of benefit, or other reasons. However, other studies have reported some important beneficial effects (**Table 2**). Some of those trials are described briefly in the following paragraphs.

**Table 2:** Clinical trials of ICIs plus metabolic targets showing promising results.

Clinical Trial No.	Metabolic Target	Drug	ICI	Cancer Type	Study Status	Results
NCT01604889	IDO1	Epacadostat	Ipilimumab	Metastatic Melanoma	Terminated	Drug well tolerated in patients with advanced melanoma
NCT03894540	Glutaminase	IPN60090	Pembrolizumab	Advanced Solid Tumor	Terminated	Drug well tolerated at biologically active doses; preliminary antitumor activity observed
NCT02903914	Arginase	CB-1158	Pembrolizumab	Advanced/Metastatic Solid Tumors	Recruitment Completed	Increase in plasma arginine and intratumoral CD8^+^ T cells
NCT03618654	AMPK	Metformin	Durvalumab	Head and Neck Squamous Cell Carcinoma	Active	Decrease in cellular density of FOXP3^+^ cells; increase in CD8+ T cell density in tumor adjacent stroma between patients receiving durvalumab and metformin versus durvalumab alone. Greater CD8^+^ –FOXP3+ intercellular distances associated with pathologic response
NCT02503774	Adenosine	Anti-CD73 Drug MEDI9447	Durvalumab (MEDI4736)	Advanced Solid Tumors	Active	Antitumor activity reported in EGFRm NSCLC; objective response was durable in immunotherapy resistant tumor types
NCT02754141	Adenosine	BMS-986179	Nivolumab	Malignant Solid Tumors	Recruitment Completed	Drug well tolerated; combination therapy showed preliminary antitumor activity
NCT02655822	Adenosine	Ciforadenant	Atezolizumab	Renal Cell Cancer Metastatic Castration Resistant Prostate Cancer	Recruitment Completed	Increased recruitment of CD8^+^ T cells into tumor; broadened circulating T-cell repertoire
NCT03829436	PPARα	TPST-1120	Nivolumab	Advanced Solid Tumors	Active	Objective responses observed from combination therapy in subjects previously refractory to anti-PD1 therapy; 2/2 responders in late-line RCC, 1 responder with heavily pretreated CCA (tumors generally not responsive to anti-PD1 alone)

ICI, immune checkpoint inhibitor; EGFRm, epidermal growth factor receptor mutation; NSCLC, non-small cell lung cancer; RCC, renal cell carcinoma; CCA, cholangiocarcinoma.

Arginine is an amino acid required for T-cell activation and proliferation. In the TME, MDSCs consistently secrete arginase, which depletes arginine levels within the tumor, leading to impaired T-cell proliferation and cytokine production (235). CD1158, a molecular inhibitor of arginase, was reported to increase plasma arginine when used in combination with pembrolizumab in an ongoing clinical trial for patients with colorectal carcinoma (NCT02903914). This trial reported an increase in intratumoral CD8+ T cells in patients with microsatellite-stable colorectal cancer after treatment with CB1158 and pembrolizumab.

Metformin is commonly prescribed for the treatment of type II diabetes. Metformin has also been shown to downregulate cytosolic oxidative phosphorylation by inhibiting complex I of the electron transport chain, which ordinarily has an important role in tumor growth (218, 236, 237). Numerous preclinical, epidemiological, and clinical studies have suggested that metformin can inhibit cancer cell growth and proliferation (238). An ongoing phase I trial (NCT03618654) investigating the effect of metformin in combination with durvalumab for head and neck squamous cell carcinoma reported a significant decrease in FOXP3 T_regs_ and an increase in CD8+ cellular density in the stroma adjacent to the tumor. Greater increases in CD8+ cell density and decreases in FOXP3 cell density were observed in patients receiving metformin with durvalumab relative to durvalumab alone (239). These findings were supported by a retrospective review of patients given FDA-approved nivolumab, pembrolizumab, or atezolizumab, with or without metformin, for newly diagnosed stage IV non-small cell lung cancer (NSCLC). This study reported improved clinical outcomes (overall response rate, disease control rate, overall survival) among patients who received an ICI with metformin; however, this observed improvement was not statistically significant (240). Several clinical trials are currently underway to test metformin with anti-PD1 ICIs. In one such trial sponsored by Northwestern University (NCT03048500), the safety, tolerability, and antitumor efficacy of a metformin-nivolumab combination are being evaluated in patients with NSCLC with or without prior treatment with PD1/PDL1 inhibitors. Another phase IB clinical trial, conducted at Okayama University in Japan (UMIN000028405), is testing the safety, efficacy, and pharmacokinetics of a nivolumab–metformin combination for refractory/recurrent tumors. Another phase II trial (NCT03800602) is underway to evaluate the effects of combining metformin with nivolumab on overall response rate among patients with microsatellite-stable stage IV colorectal cancer that has not responded to previous treatment. Finally, an investigator-initiated phase I clinical trial (NCT03311308) is currently evaluating the effectiveness and safety of pembrolizumab with metformin for advanced-stage melanoma.

Adenosine is produced mainly from ATP catabolism mediated by CD39 and CD73; adenosine binds to adenosine receptors to trigger downstream signaling (142). Adenosine concentrations are significantly elevated in some solid tumors. An elevated level of adenosine in the TME can potentially impair T-cell function by inducing the accumulation of intracellular cAMP (241). The anti-CD73 agent MEDI9447 combined with the anti-PDL1 agent durvalumab is currently being investigated for patients with advanced colorectal or pancreatic cancer (NCT02503774). Also, combinations of the anti-CD73 antibody BMS-986179 and nivolumab are being investigated for the treatment of various advanced solid tumors (NCT02754141). Early findings from the latter study reported that the combination therapy had a more pronounced antitumor effect compared with ICI alone.

Ciforadenant, a molecule targeting the adenosine-A2A receptor on T lymphocytes and other immune cells, may also enhance the antitumor activity of T cells. A clinical trial investigating ciforadenant (NCT02655822) reported that it was well tolerated, alone and in combination with atezolizumab, among patients with advanced metastatic castration-resistant prostate cancer, with 5 of 33 such patients showing had tumor regression (241). This combination has also shown antitumor activity against renal cell carcinoma and NSCLC.

Another adenosine A2a receptor antagonist (AZD4635), given as monotherapy or in combination with durvalumab, was also found to be well tolerated and to have clinical benefit among patients with refractory metastatic castrate-resistant prostate cancer (NCT02740985). Tumor responses were noted in 2 of 39 patients given AZD4635 as monotherapy, and 6 of 37 given combination therapy; prostate-specific antigen responses were found in 3 of 60 patients (monotherapy) and 10 of 45 patients (combination therapy) (242). Several other clinical trials of adenosine antagonists are currently underway, among them a phase II study of the safety and efficacy of AZD4635 with durvalumab or oleclumab for patients with prostate cancer (NCT04089553); and a nonrandomized phase II study of the efficacy and safety of AZD4635 plus durvalumab ± cabazitaxel for patients with progressing advanced prostate cancer (NCT04495179). Finally, the efficacy of a dual adenosine receptor antagonist (AB928) and a PD1 checkpoint inhibitor (AB122) is being tested in combination with short-course radiotherapy and consolidation chemotherapy for patients with rectal cancer (NCT05024097).

Another clinical trial approach currently being evaluated targets the regulation of lipid metabolism and FAO. As noted previously, tumor cells rapidly proliferate, metastasize, quickly utilize existing glucose stores, and undergo metabolic reprogramming shifts toward FAO. Fatty acids in turn support the metabolism of suppressive immune cells in the TME in addition to tumor growth. PPARα is a ligand-activated nuclear transcription factor that regulates lipid metabolism and FAO (243). TPST-1120, a selective PPARα antagonist, blocks the transcription of PPARα target genes, leading to an intracellular metabolism shift from FAO to glycolysis (244). Reduction of fatty acids in the TME leads to the direct killing of tumor cells that depend on FAO and skews macrophages from the immunosuppressive M2 phenotype to an effector M1 phenotype, thereby improving the cytotoxicity of immune effector cells (245). Early findings from an ongoing trial of TPST-1120 in combination with nivolumab have shown that TPST-1120 is well-tolerated, both as a single agent and in combination with nivolumab, and the combination has shown promising objective responses among subjects with disease previously refractory to anti-PD1 therapy, including 2 of 2 responders with pretreated renal cell carcinoma, and a subject with heavily pretreated cholangiocarcinoma, a tumor that generally does not respond to anti-PD1 alone.

Other clinical trials currently underway are targeting different metabolic pathways. One example is a trial of DON, a glutamine antagonist that suppresses cancer cell metabolism but concurrently enhances the metabolic fitness of tumor CD8+ T cells. DRP-104, a peptide prodrug of DON, is as effective as DON in inhibiting tumor growth but has markedly less toxicity. An ongoing clinical trial (NCT04471415) is currently assessing the safety, tolerability, pharmacokinetics, pharmacodynamics, and preliminary antitumor activity of DRP-104, given as single-agent therapy.

These clinical trial results support the contention that metabolic pathways can be exploited for therapeutic purposes. That said, we urge caution in interpreting these findings, as many of these clinical trials are still ongoing and the findings described here are based on journal and conference proceedings with limited sample sizes. More studies are needed to obtain reliable information on the agents’ safety, tolerability, exposure duration, and pharmacokinetics, especially when they are used in combination with chemotherapy or radiation in attempts to improve treatment efficacy. Nevertheless, increasing interest in developing various metabolic targets to enhance antitumor T-cell activity or interfere with aberrant tumor metabolic pathways is a promising step towards designing more effective immunotherapy strategies.

## Author contributions

Concept developed by MC and SG. SG: writing–review and editing. PG: writing–review. HC: writing–review. SN: writing–review. TR: writing–review. LD: writing–review. HB: writing–review. FM: writing–review. HJ: writing–review. JW: writing–review. MC: writing–review and editing. All authors have read approved the final version of this manuscript. All authors contributed to the article.
